# Nuclear Envelope Protein Lem2 is Required for Mouse Development and Regulates MAP and AKT Kinases

**DOI:** 10.1371/journal.pone.0116196

**Published:** 2015-03-19

**Authors:** Olga Tapia, Loren G. Fong, Michael D. Huber, Stephen G. Young, Larry Gerace

**Affiliations:** 1 Department of Cell and Molecular Biology, The Scripps Research Institute, 10550 N. Torrey Pines Rd., La Jolla, California 92037, United States of America; 2 Department of Medicine, University of California Los Angeles, Los Angeles, California 90095, United States of America; 3 Department of Human Genetics, University of California Los Angeles, Los Angeles, California 90095, United States of America; University of Toronto, CANADA

## Abstract

The nuclear lamina, along with associated nuclear membrane proteins, is a nexus for regulating signaling in the nucleus. Numerous human diseases arise from mutations in lamina proteins, and experimental models for these disorders have revealed aberrant regulation of various signaling pathways. Previously, we reported that the inner nuclear membrane protein Lem2, which is expressed at high levels in muscle, promotes the differentiation of cultured myoblasts by attenuating ERK signaling. Here, we have analyzed mice harboring a disrupted allele for the Lem2 gene (*Lemd2*). No gross phenotypic defects were seen in heterozygotes, although muscle regeneration induced by cardiotoxin was delayed. By contrast, homozygous *Lemd2* knockout mice died by E11.5. Although many normal morphogenetic hallmarks were observed in E10.5 knockout embryos, most tissues were substantially reduced in size. This was accompanied by activation of multiple MAP kinases (ERK1/2, JNK, p38) and AKT. Knockdown of Lem2 expression in C2C12 myoblasts also led to activation of MAP kinases and AKT. These findings indicate that *Lemd2* plays an essential role in mouse embryonic development and that it is involved in regulating several signaling pathways. Since increased MAP kinase and AKT/mTORC signaling is found in other animal models for diseases linked to nuclear lamina proteins, *LEMD2* should be considered to be another candidate gene for human disease.

## Introduction

The nuclear envelope (NE) is a specialized domain of the ER that contains inner (INM) and outer (ONM) nuclear membranes joined at nuclear pore complexes and lined by the nuclear lamina (reviewed in [1–3]). The lamina is a filamentous protein meshwork that contains a polymeric assembly of nuclear lamins, type V intermediate filament proteins found in all metazoans (reviewed in [4–6]). Three major subtypes of lamins are expressed in most differentiated mammalian somatic cells: lamins A/C, which are alternatively spliced products of the same gene, and lamin B1 and lamin B2, which arise from separate genes. The NE also includes a host of minor protein components, particularly transmembrane proteins of the INM (reviewed in [7,8]). Although the ONM is continuous with more peripheral ER, many transmembrane proteins are highly concentrated at the INM, partly due to their interactions with lamins and/or chromatin (reviewed in [9]). Some of these transmembrane proteins have been characterized in detail, including the lamin B receptor (LBR), emerin, Lamina-Associated Polypeptide 1 (LAP1), LAP2, and MAN1 [7,10]. The nuclear lamina is involved in organizing the structure of the NE, attaching chromatin to the INM, modulating interphase chromosome structure, and anchoring the cytoplasmic cytoskeleton to the nucleus [3,10]. These functions involve the polymeric nuclear lamin core as well as integral and peripheral membrane proteins associated with nuclear membranes.

At least 15 human diseases are caused by mutations in proteins associated with the NE (reviewed in [1,11]). The diseases, termed “laminopathies” or “nuclear envelopathies,” most commonly arise from mutations in the gene for lamins A/C (*LMNA*). They include various forms of muscular dystrophy, cardiomyopathy, lipodystrophy, neuropathy, and premature aging syndromes. Laminopathies also have been linked to mutations in transmembrane proteins of the NE. For example, mutations in the gene for emerin (*EMD*) cause Emery-Dreifuss muscular dystrophy (EDMD)[12], and mutations in the *LEMD3* gene encoding MAN1 cause sclerosing bone dysplasias [13]. Although mutations in NE proteins have been associated with defects in signaling, gene expression, and NE/nuclear structure [2,11], the proximal molecular mechanisms leading to human disease are largely unclear.

In mammals, emerin and MAN1, along with the INM proteins LAP2β and Lem2, contain a LEM homology domain [14]. The LEM domain is an ~40-amino acid sequence that binds to a dimer of BAF, a small polypeptide involved in chromatin organization [7]. LEM domain proteins have a widespread tissue distribution, although their expression levels vary. Studies in cultured cells and animal models have suggested a diverse range of functions for LEM domain proteins of the INM, including regulation of signaling and chromatin structure [7], and modulation of NE reassembly at the end of mitosis [15]. Some of the most extensive insights have been obtained for MAN1, which is involved in attenuating TGF- signaling [13,16,17]. MAN1 interacts directly with the phosphorylated forms of Smad 2/3 [16,17] and with a Smad phosphatase [18], and might provide a scaffold that facilitates Smad dephosphorylation. Mice with a gene-trap allele of *Lemd3* encoding MAN1 die at midgestation with a defect in vasculogenesis associated with overactive TGF-β[19,20]. Deficiency of emerin in various experimental models has been associated with elevated ERK signaling [21,22]. Although mice lacking emerin expression appear phenotypically normal [23], the absence of emerin in mice enhanced the muscular dystrophy-like disorder associated with LAP1 deficiency [24]. Multiple LEM domain proteins are found in other metazoans and have been linked to tissue-specific pathology in *C*. *elegans* [25], *Drosophila* [26], and *Xenopus* [27,28]. While different LEM domain proteins clearly have distinctive functions, functional overlap also has been reported [26,27,29,30].

Lem2, which we identified in a proteomics screen for novel NE transmembrane proteins [31], is related to MAN1 in overall membrane topology [32]. Both proteins contain N- and C-terminal nucleoplasmic domains flanking a central region with two transmembrane sequences and a luminal segment. Moreover, both proteins share sequence homology in the luminal domain and in the second nucleoplasmic domain [32], although Lem2 lacks the Smad-binding region found in MAN1 [33]. Lem2 is expressed widely and binds to A-type lamins [32,34], and RNAi-mediated knockdown of Lem2 in certain cell types leads to irregular nuclear structure and reduced proliferation [35]. We found that Lem2 is strongly upregulated during myoblast differentiation [34] and plays a critical role in attenuating ERK signaling during this process [29]. Here, we have analyzed the functions of *Lemd2* in mouse with a gene-trap allele (*Lemd2* ^*Gt*^). While *Lemd2* ^*+/Gt*^ mice were almost normal phenotypically, *Lemd2* ^*Gt/Gt*^ mice exhibited embryonic lethality by E11.5. At E10.5, there was defective growth of most embryonic regions, along with substantially increased activation of MAP kinases and AKT in embryonic extracts. Similar signaling defects were observed with Lem2 knockdown in cultured cells. Together, these results reveal that Lem2 plays a crucial role in mouse embryonic development and in the regulation of several signaling pathways, underscoring the importance of this protein in mammals.

## Results

### Derivation and characterization of mice with a *Lemd2* gene-trap allele

**Fig 1:**
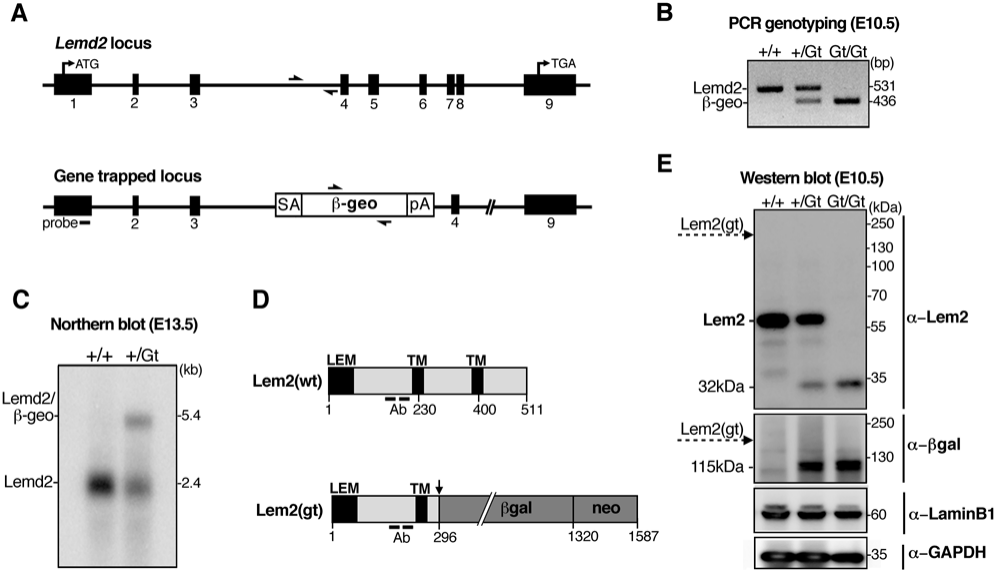
Gene-trap disruption of the *Lemd2* locus. (**A**) Upper panel: Schematic of the *Lemd2* gene, showing exon organization (black boxes). Lower panel: Depiction of the gene-trap insertion in the *Lemd2* gene (see text). The location of the RNA probe used for Northern blotting (“probe”) and the positions of the primers used for PCR genotyping are indicated. (**B**) PCR analysis of yolk sac DNA from *Lemd2* ^+/+^, *Lemd2* ^+/Gt^, and *Lemd2* ^Gt/Gt^ embryos. (**C**) Northern blot analysis of mRNA from wild-type and heterozygous embryos at E13.5. (**D**) Schematic of the wild-type Lem2(wt) protein, with the LEM domain and transmembrane (TM) sequences indicated, and of the Lem2(gt) predicted fusion protein. ‘Ab’ indicates the peptides used for production and purification of anti-Lem2 antibodies. (**E**) Western blot analysis of E10.5 embryo extracts with the indicated antibodies. Dotted arrow indicates the predicted position of the Lem2(gt) fusion protein and the 32-kDa mark designates the putative Lem2 fragment.

We used mouse embryonic stem cells with a gene-trap insertion in the *Lemd2* locus (*Lemd2* ^*Gt*^) to derive *Lemd2* knockout mice (Fig. 1). The gene trap introduced a sequence between exons 3 and 4 of *Lemd2* that contains a splice acceptor site (SA), a cDNA encoding the β-galactosidase reporter linked to neomycin (βgeo), and a SV40 cleavage/polyadenylation site (pA) (Fig. 1A). PCR analysis of genomic DNA was used for genotyping (Fig. 1B). To characterize transcripts of the wild-type and gene-trap alleles, we performed Northern blot analysis on mRNA from E13.5 embryos with an oligonucleotide probe recognizing exon 1 of *Lemd2* (Fig. 1C). A single band corresponding to the 2.4 kb *Lemd2* mRNA was detected in wild-type embryos (Fig. 1C, lane 1). In *Lemd2* ^*+/Gt*^ embryos (Fig. 1C, lane 2), a 5.4 kb band also was observed, matching the size of the predicted *Lemd2-βgeo* transcript. These results, combined with our analysis of *Lemd2* ^*Gt*^ protein products at various developmental stages (described below), indicate that “splicing around” the insertional mutation (to downstream *Lemd2* exons) does not occur at significant levels.

Wild-type *Lemd2* produces a 511-amino acid protein, but the gene-trap *Lemd2-βgeo* allele is predicted to generate an ~175-kDa fusion protein, “Lem2(gt)”, containing the N-terminal 296 amino acids of Lem2 fused to the β-galactosidase-*neo* fusion (*βgeo*) (Fig. 1D). Affinity-purified antibodies against Lem2 were used to analyze extracts from E8.5, E10.5, and E13.5 embryos by western blotting (Fig. 1E and S1A Fig.). Expression of Lem2 was detected at all three stages, with a progressive increase from E8.5 to E13.5. In wild-type embryos, the antibodies detected a single Lem2 band at the expected molecular weight (58 kDa). This band was reduced in intensity by about one-half in *Lemd2* ^*+/Gt*^ extracts and was absent from *Lemd2* ^*Gt/Gt*^ extracts (obtained only at E8.5 and E10.5). Unexpectedly, the predicted 175-kDa Lem2*-βgeo* fusion protein was not detected in either *Lemd2* ^*+/Gt*^ or *Lemd2* ^*Gt/Gt*^ extracts with antibodies against either Lem2 or β-galactosidase. Instead, anti-Lem2 recognized a 32-kDa band, and anti-β-galactosidase recognized a 115-kDa band (Fig. 1E, top and middle panels). The 32-kDa and 115-kDa polypeptides likely represent degradation products of the predicted Lem2*-βgeo* fusion protein, with the 32-kDa band containing sequences from Lem2 exons 1–3 and the 115-kDa species containing β-galactosidase. In *Lemd2* ^*+/Gt*^ embryos, the level of the presumptive Lem2 exons 1–3 product was only ~1/4–1/3 that of the 58-kDa Lem2 band (S1A and C Fig.), raising the possibility that the 32-kDa Lem2 fragment might be unstable. Consistent with that idea, the 32-kDa band could not be detected in skeletal muscle from adult *Lemd2* ^*+/Gt*^ animals (S1B Fig.), although the 115-kDa β-galactosidase band was clearly evident. The loss of Lem2 in E10.5 embryos with the *Lemd2* ^*Gt*^ allele did not affect the levels of lamin B1, which is expressed throughout embryogenesis (Fig. 1E) [6]. Furthermore there was no consistent change in the levels of emerin (data not shown). Lamins A/C are not expressed until E12 [36], well after the onset of Lem2 expression.

**Fig 2:**
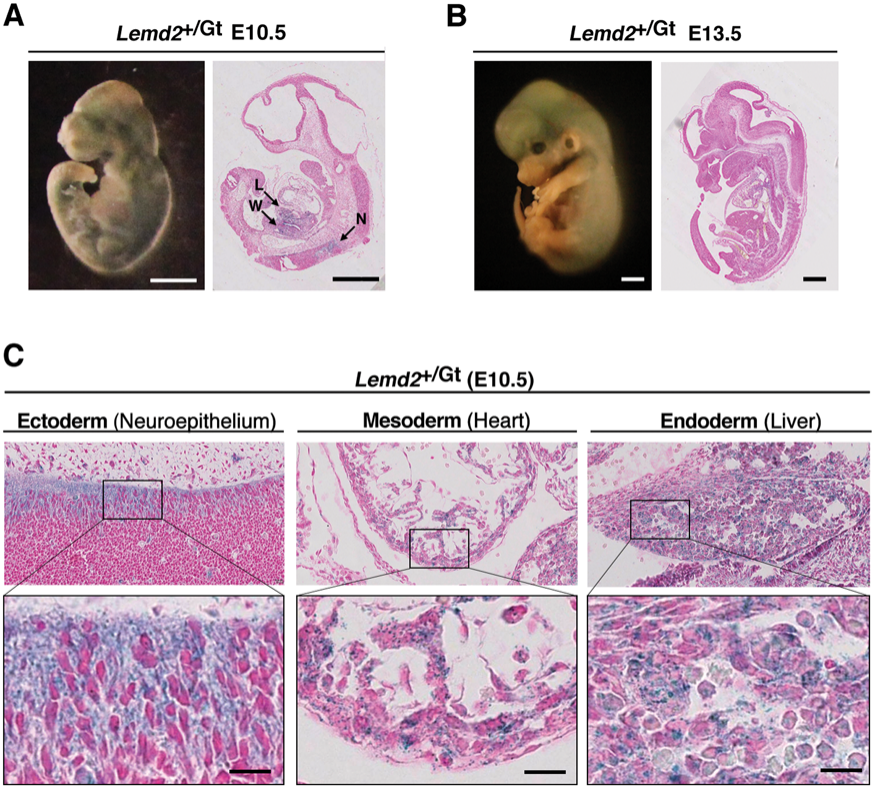
Lem2 expression during embryonic development. (**A-B**) Whole mount lateral views (left panels) and sagittal sections (right panels) of *Lemd2* ^*+/Gt*^ embryos at E10.5 (**A**) and E13.5 (**B**) stained with X-gal. Arrows in (B) indicate tissues with slightly higher X-gal staining: L, liver; W, Wolffian duct; N, neuroepithelium. Bars: 1 mm. (**C**) Sagittal sections of the indicated regions of a *Lemd2* ^*+/Gt*^ embryo at E10.5 stained with X-gal and Nuclear Fast Red. Lower panels are high-magnification views of boxed areas. Bars: 15 μm.

Lem2 is widely expressed in different adult human and mouse tissues [32,34], although levels are much greater in cardiac and skeletal muscle [34]. We analyzed heterozygous *Lemd2* ^*+/Gt*^ embryos by X-gal staining to examine the expression pattern of *Lemd2* ^*Gt*^ at midgestation (Fig. 2). Analysis of whole-mount embryos at E10.5 and E13.5 (Fig. 2A, B, left panels) showed variable levels of X-gal staining throughout the embryo. Examination of histological sections at low magnification (Fig. 2A, B, right panels) revealed only weak labeling, although slightly higher staining sometimes was seen in the developing liver, Wolffian duct and certain areas of neuroectoderm (Fig. 2A, right, arrows). In sections from E10.5 embryos viewed at higher magnification (Fig. 2C), X-gal staining was detected in cells arising from all three germ layers, as exemplified by views of neuroepithelium (ectoderm), heart (mesoderm), and liver (endoderm). From these results, we conclude that Lem2 is expressed as early as E8.5 during embryogenesis and is present in all three germ layers by E10.5. Also, *Lemd2* ^*Gt/+*^ embryos contain reduced levels of a Lem2-related fragment rather than the full-length gene-trap fusion protein; this Lem2 fragment is not detectable in adult skeletal muscle.

### Homozygous disruption of Lemd2 causes embryonic lethality by E11.5

*Lemd2* ^*+/Gt*^ mice were viable, developmentally normal and fertile, and showed no abnormalities in growth or survival for up to one year (S1D Fig. and data not shown). Histological analyses of cardiac and skeletal muscle from *Lemd2* ^*+/+*^ and *Lemd2* ^*+/Gt*^ mice at 1, 4, and 8 months revealed no differences in overall morphology and skeletal muscle fiber diameter (S1E Fig. and data not shown). Also, neither genotype showed evidence of skeletal muscle fibrosis (S1E Fig.). However, there were modest kinetic differences in muscle regeneration in *Lemd2* ^*+/Gt*^ animals (see below).

**Fig 3:**
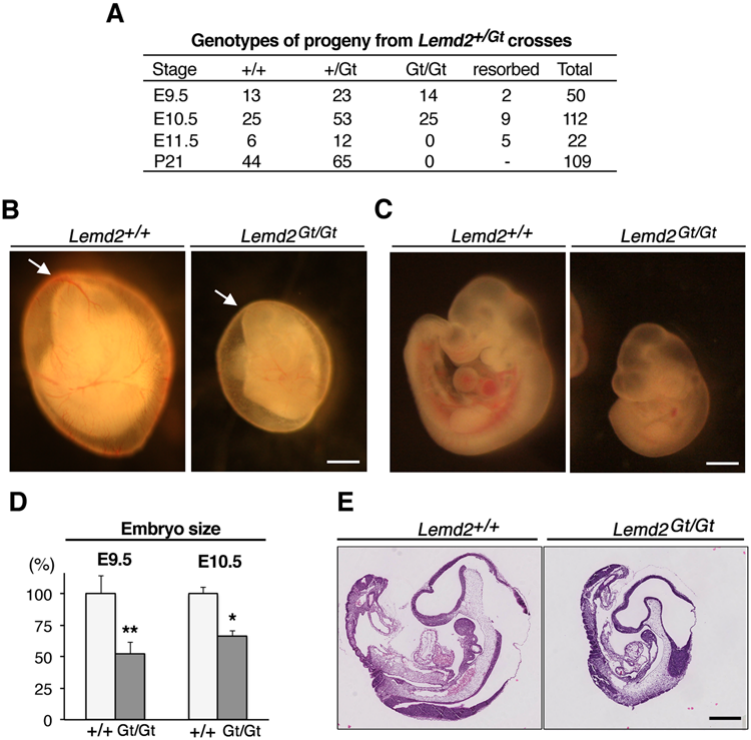
Abnormal growth and embryonic lethality of *Lemd2* ^Gt/Gt^ mice. (**A**) Genotypes of embryos and pups produced from intercrossing of *Lemd2* ^*+/Gt*^ animals. (**B, C**) Bright-field images of *Lemd2* ^*+/+*^ (left) and *Lemd2* ^*Gt/Gt*^ (right) embryos at E10.5. (**B**) Views of embryos within yolk sacs. (**C**) Views of embryos with yolk sacs removed. (**D**) Crown-to-rump length of embryos at E9.5 and E10.5. *Lemd2* ^*+/+*^ *n* = 6; *Lemd2* ^*Gt/Gt*^ *n* = 5. Data are presented as mean ± SD. (*p < 0.0005, **p < 0.005). (**E**) Sagittal sections of *Lemd2* ^*+/+*^ and *Lemd2* ^*Gt/Gt*^ E10.5 embryos stained with H&E. Bars: 500 μm.

Heterozygotes were intercrossed to obtain *Lemd2* ^*Gt/Gt*^ animals. Of 109 live-born offspring, 44 were *Lemd2* ^*+/+*^, 65 were *Lemd2* ^*+/Gt*^, and none were *Lemd2* ^*Gt/Gt*^ (Fig. 3A). Analysis by the Chi-squared test indicates that *Lemd2* ^*Gt/Gt*^ embryos die before birth (*p* = 8 × 10^-8^). The ratio of *Lemd2* ^*+/+*^ to *Lemd2* ^*+/Gt*^ mice (44:65) did not match a perfect Mendelian ratio (1:2), but it is important to note that the *p*-value for the observed ratio was not statistically different from the 1:2 ratio (*p* = 0.1193).

We carried out timed matings of *Lemd2* ^*+/Gt*^ mice to identify the stage when *Lemd2* ^*Gt/Gt*^ embryos die (Fig. 3A). Genotyping of 50 embryos at E9.5 and 112 embryos at E10.5 revealed a normal Mendelian ratio of *Lemd2* ^*Gt/Gt*^ embryos (Fig. 3A), although some embryos undergoing resorption could not be genotyped. In contrast, no *Lemd2* ^*Gt/Gt*^ embryos were observed at E11.5. These results indicate that *Lem2*-deficient mice die between E10.5 and E11.5.

To investigate the basis for the lethality of *Lemd2* ^*Gt/Gt*^, we carried out more detailed analyses of E10.5 embryos (Fig. 3). The *Lemd2* ^*Gt/Gt*^ embryos were conspicuously smaller than *Lemd2* ^*+/+*^ and *Lemd2* ^*Gt/+*^ littermates, had a paler yolk sac, and appeared to contain less blood, as judged by bright-field microscopy (Fig. 3B-C). Nonetheless, the great majority of *Lemd2* ^*Gt/Gt*^ embryos exhibited the morphogenetic hallmarks of E10.5 embryos, such as a beating heart, eyes, branchial arches, forebrains, midbrains, and hindbrains, limbs, and an elongated tail with obvious somites. A small fraction (~10%) of *Lemd2* ^*Gt/Gt*^ embryos had more severe defects, particularly abnormalities in craniofacial development (S2 Fig.).

**Fig 4:**
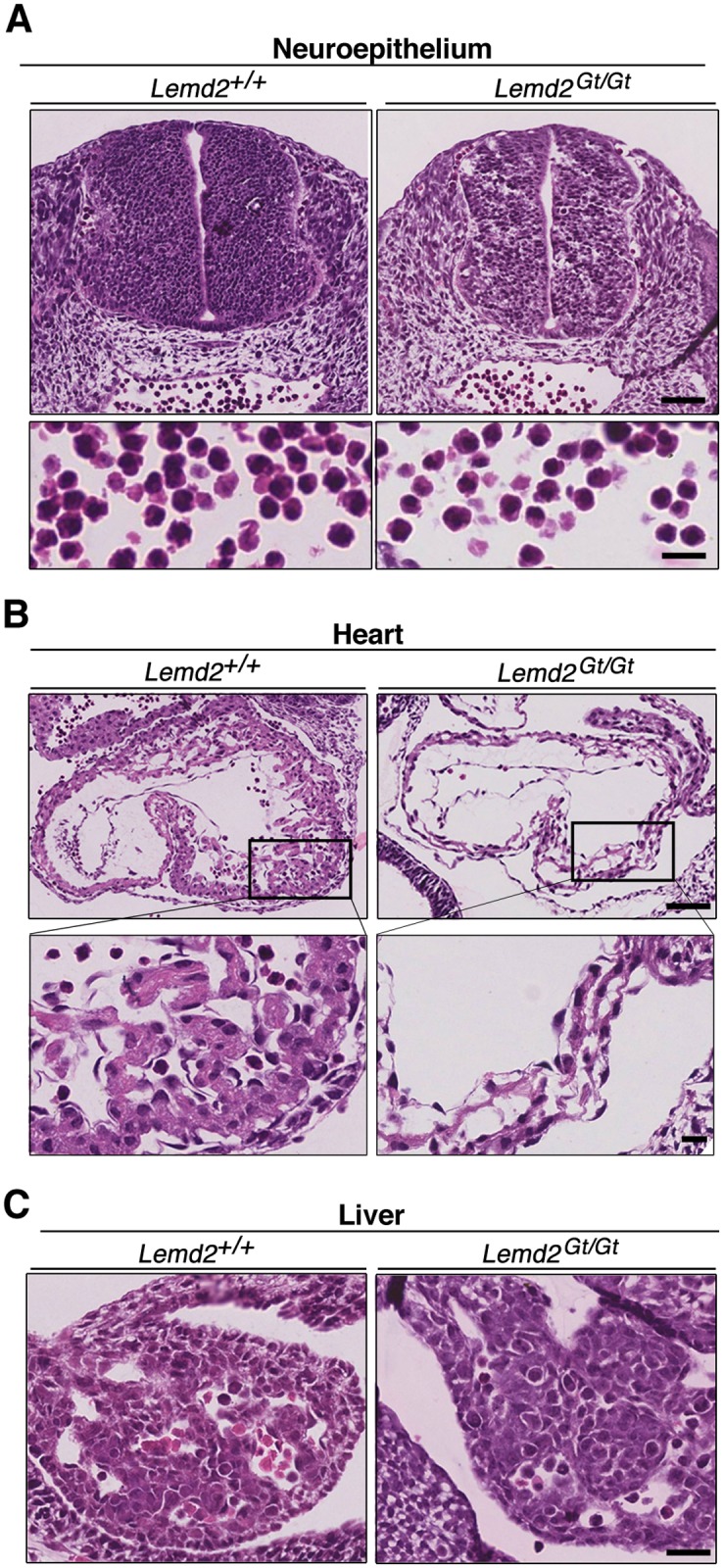
Impaired neurogenesis and cardiogenesis in *Lemd2* ^Gt/Gt^ embryos. (**A-C**) Histological sections of *Lemd2* ^*+/+*^ (left) and *Lemd2* ^*Gt/Gt*^ (right) E10.5 embryos stained with H&E. (**A**) Top panels: transverse sections of the posterior region of the neural tube. Lower panels: higher magnification views of blood cells from panel A. (**B**) Sagittal sections of heart. Lower panels are high-magnification views of boxed areas. (**C**) Sagittal sections of liver. Bars: A and B top, 50 μm; A and B bottom and C, 15 μm.

Histological analyses of embryos at E10.5 revealed that organogenesis in *Lemd2* ^*Gt/Gt*^ embryos occurred in a manner similar to wild-type littermates. The mutant embryos developed heart, lungs, liver, eye structures, somites, pharyngeal arches, and primitive red blood cells (Fig. 3E, S3 Fig. and Fig. 4A). The appearance of many tissues in *Lemd2* ^*Gt/Gt*^ embryos, such as the liver (Fig. 4C), was similar to that of wild-type embryos. However, certain tissues in *Lemd2* ^*Gt/Gt*^ embryos such as neural and heart structures appeared to be less developmentally advanced and/or abnormal (Fig. 4A, B). The neuroepithelium in the neural tube (Fig. 4A, upper) and mesenchyme (S3 Fig.) had reduced cell density and many gaps between cells. Although serial sections revealed normal looping and formation of a four-chambered heart in *Lemd2* ^*Gt/Gt*^ embryos (Fig. 4B and data not shown), the myocardium was abnormally thin (only 1–3 cells thick), and the trabeculae were underdeveloped (Fig. 4B, enlarged insets).

**Fig 5:**
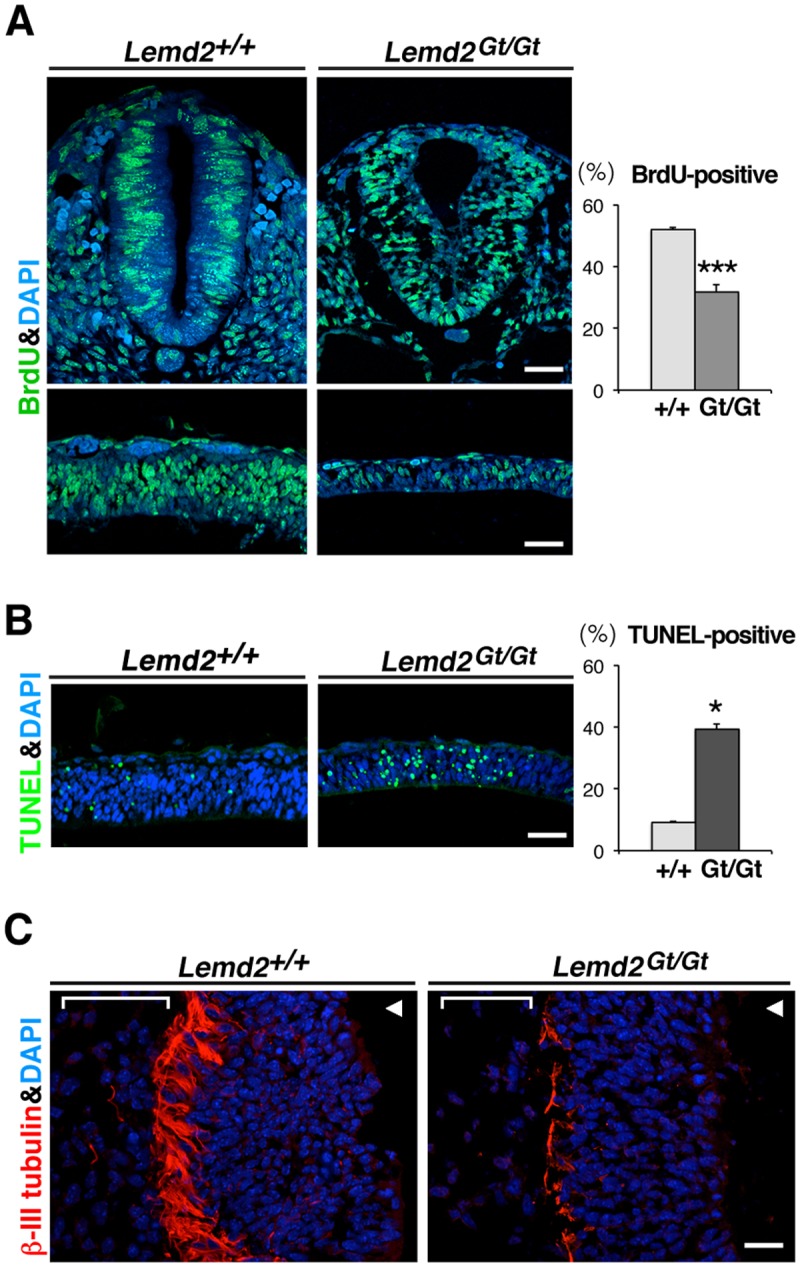
Increased apoptosis, reduced cell proliferation and smaller number of differentiated neurons in the absence of Lem2. (**A**) Cell proliferation monitored by BrdU incorporation in *Lemd2* ^*+/+*^ and *Lemd2* ^*Gt/Gt*^ embryos at E10.5. Anti-BrdU staining (green) with DAPI counterstaining (blue) of cryosections of the neural tube, showing transverse (upper panels, posterior region) and sagittal (lower panels, midbrain region) views. Bar graph: Quantification of the percentage of BrdU-positive cells in the neuroepithelium of *Lemd2* ^*+/+*^ and *Lemd2* ^*Gt/Gt*^ E10.5 embryos. (**B**) Midbrain sections adjacent to those of Fig. 5A (lower panels) subjected to terminal dUTP nick end labeling (TUNEL) analysis (green) and DAPI counterstaining (blue). Bar graph: Quantification of the percentage of TUNEL-positive cells in the midbrain of *Lemd2* ^*+/+*^ and *Lemd2* ^*Gt/Gt*^ E10.5 embryos. (**C**) Immunofluorescence staining of class III β-tubulin (red) and DAPI counterstaining (blue) in the hindbrain neuroepithelium of wild-type and *Lemd2* ^*Gt/Gt*^ embryos at E10.5. Arrowheads point to the ventricular surface of the neuroepithelium, and brackets indicate the layer of mesenchyme. Data are presented as mean ± SD. (*p < 0.0005, *** *p* < 0.05). Bars: 50 μm.

We analyzed neural tissues of E10.5 *Lemd2* ^*Gt/Gt*^ embryos for cell growth abnormalities, given that these regions had the highest *Lemd2* expression by X-gal staining. To investigate cell proliferation, we carried out *in vivo* labeling with BrdU. The neural tube from wild-type embryos had BrdU incorporation in ~50% of the cells (Fig. 5A). In contrast, the same region from littermate *Lemd2* ^*Gt/Gt*^ embryos showed BrdU incorporation in only ~20% of the cells (Fig. 5). We also evaluated apoptosis using the TUNEL assay. Quantitative analysis of TUNEL staining in midbrain neuroepithelium revealed 7-fold higher apoptosis in *Lemd2* ^*Gt/Gt*^ embryos than in wild-type littermate embryos (Fig. 5B, right panel). Finally, we analyzed neural tissue for differentiation by immunofluorescence staining of cryosections with antibodies against class-III β-tubulin, a widely used marker of postmitotic neurons [37] (Fig. 5C). Wild-type embryos showed a prominent layer of cells containing neuronal β-tubulin along the basal surface of the neuroepithelium (Fig. 5C, left panel). This band of class-III β-tubulin-positive cells was markedly reduced in *Lemd2* ^*Gt/Gt*^ embryos (Fig. 5C, right). These observations suggest that the lack of Lem2 in E10.5 *Lemd2* ^*Gt/Gt*^ embryos leads to reduced proliferation of the neuroepithelium, increased apoptosis in neural progenitor cells, and impaired accumulation of differentiated neurons.

### Hyperactivation of MAPK and AKT signaling in *Lemd2* ^*Gt/Gt*^ embryos

**Fig 6:**
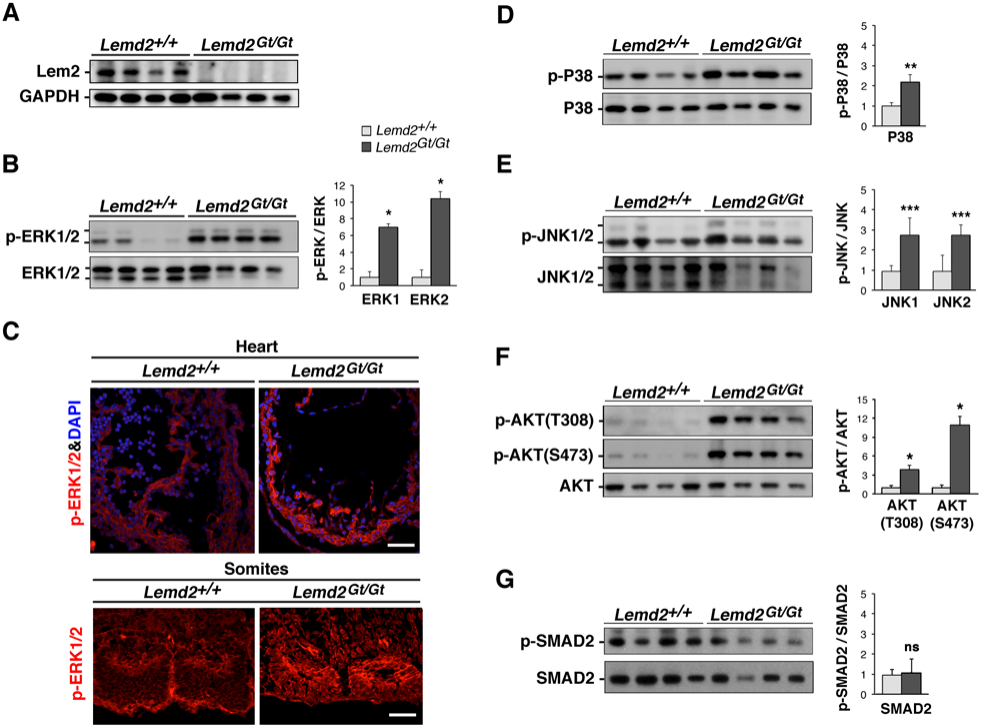
Activation of MAP kinase and AKT signaling pathways in *Lemd2*-deficent embryos. (**A, B, D-G**) Western blot analysis of protein extracts from *Lemd2* ^*+/+*^ and *Lemd2* ^*Gt/Gt*^ embryos at E10.5. Samples from 4 separate embryos of each genotype are shown. All extracts were from embryos with beating hearts. (**A**) Labeling with anti-Lem2 and anti-GAPDH. (**B, D-G**) Labeling with antibodies to the phosphorylated activation sites of various kinases or kinase effectors (upper panels) and to nonphosphorylated epitopes of the corresponding proteins (lower panels). (**B**) Anti-ERK1/2. (**D**) Anti-p38. (**E**) Anti-JNK1/2. (**F**) Anti-AKT. (**G**) Anti-Smad2. Error bars indicate standard deviations (*p < 0.0005, **p < 0.005, ***p < 0.05, ns “not significant”). (**C**) Immunofluorescence staining of sagittal cryosections of *Lemd2* ^*+/+*^ and *Lemd2* ^*Gt/Gt*^ hearts and somites at E10.5 with anti-phospho-ERK1/2 antibodies. All images were captured and displayed with exactly the same parameters.

We previously found that knockdown of Lem2 in C2C12 cells leads to increased ERK activity and a blockade of myogenic differentiation [29]. To determine if the embryonic lethality of *Lemd2* ^*Gt/Gt*^ mice is associated with aberrant signaling, we analyzed the activity of several signaling pathways in E10.5 *Lemd2* ^*Gt/Gt*^ embryos by performing western blotting on embryo extracts with antibodies against phosphorylated forms of key signaling molecules. Extending our earlier results in cultured cells [29], we found that the level of ERK1/2 phosphorylation was ~7–10-fold higher in *Lemd2* ^*Gt/Gt*^ embryo extracts than in wild-type embryo extracts (Fig. 6A, B). Immunofluorescence staining of cryosections of the hearts and somites revealed increased levels of phospho-ERK1/2 in *Lemd2* ^*Gt/Gt*^ embryos (Fig. 6C). We also found an ~2–3-fold increase in the level of phosphorylation of p38 and JNK MAP kinases in *Lemd2* ^*Gt/Gt*^ embryos (Fig. 6D, E). Finally we found ~4–10-fold more phosphorylation of AKT at the Thr308 and Ser473 activation sites [38] in *Lemd2* ^*Gt/Gt*^ embryo extracts (Fig. 6F). By contrast, we found no changes in the level of phosho-Smad2, which reflects activation of TGF-β signaling [39] (Fig. 6F) and is increased in MAN1-deficient embryos [19,20]. Our results reveal hyperactivation of three major MAP kinase pathways as well as AKT in E10.5 *Lemd2* ^*Gt/Gt*^ embryos, but no change in the activation of TGF-β signaling associated with Smad2 phosphorylation.

**Fig 7:**
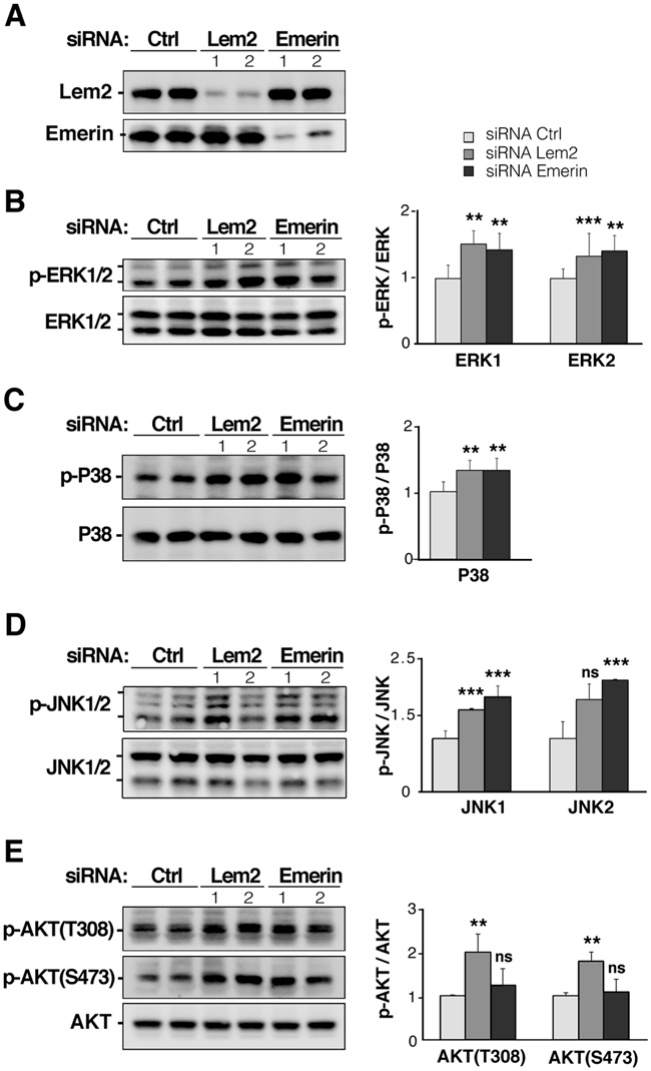
Activation of various signaling pathways in C2C12 cells by knockdown of Lem2 or emerin. (**A**) Immunoblot showing expression of Lem2 and emerin in C2C12 cells transfected with control (Ctrl) siRNA or either of two different siRNAs targeting Lem2 or emerin mRNA. (**B-D**) Western blot analysis of the phosphorylated and total levels of ERK1/2 (**B**), P38 (**C**), JNK1/2 (**D**), and AKT at Tyr307 and Ser473 phosphorylation sites (**E**). Graphs show plots of band intensities. Bars indicate the ratio of phosphorylated protein to non-phosphorylated protein normalized to Ctrl. Values are mean ± standard deviations for *n* = 4 samples per group (*p < 0.0005, **p < 0.005, ***p < 0.05, ns “not significant”).

We attempted to generate immortalized mouse embryonic fibroblasts (MEFs) from E9.5–10.5 *Lemd2* ^*Gt/Gt*^ embryos in order to further analyze signaling using a cultured cell model. Although we readily obtained immortalized MEFs from *Lemd2* ^*+/+*^ embyros, we were unsuccessful with *Lemd2* ^*Gt/Gt*^ littermates. As an alternative, we analyzed proliferating murine C2C12 myoblasts where Lem2 was depleted with siRNAs (Fig. 7A). In parallel, we examined cells with knockdown of emerin (Fig. 7A), given that emerin and Lem2 have overlapping functions in C2C12 myoblast differentiation [29] and because depletion of emerin in cultured cells was reported to activate ERK [22]. Knockdown of Lem2 did not detectably affect the level of emerin (Fig. 7A) or of other NE marker proteins we examined including LAP2β (data not shown). We found that knockdown of Lem2 led to a significant increase in the level of phosphorylation of ERK1/2, p38, and JNK kinases (Fig. 7B, C), although the fold-activation was less than in extracts of *Lemd2* ^*Gt/Gt*^ E10.5 embryos (Fig. 6). Emerin knockdown resulted in similar increases in phosphorylation of the MAP kinases (Fig. 7B, C). However, the knockdown phenotypes diverged with analyses of AKT activation. Phosphorylation of AKT on both Thr308 and Ser473 sites was increased twofold by Lem2 depletion (Fig. 7D) but was unchanged by knockdown of emerin (Fig. 7D). In summary, these results show that silencing of Lem2 in cultured cells recapitulates the increased activation of MAP kinases and AKT observed in *Lemd2* ^*Gt/Gt*^ embryos. Moreover, since AKT was activated by Lem2 depletion but not by emerin knockdown, the functions of Lem2 appear to affect a broader range of signaling pathways than emerin. These experiments can help to explain our finding that disruption of *Lemd2* in the mouse leads to embryonic lethality, whereas the loss of *Emd* has virtually no phenotype [23].

### Delayed muscle regeneration associated with decreased Lem2 levels

**Fig 8:**
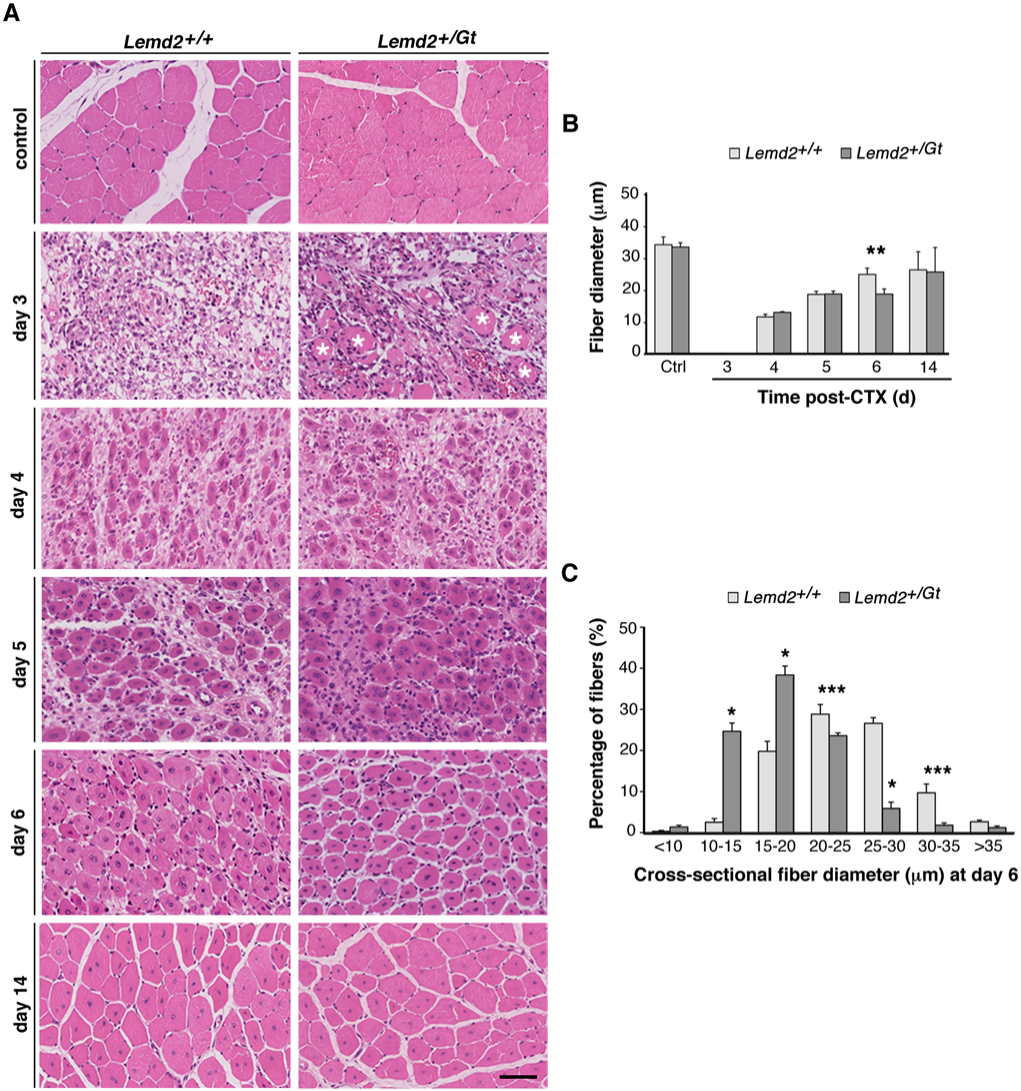
Muscle regeneration after cardiotoxin-induced injury in *Lemd2* ^+/Gt^ mice. Muscle regeneration was induced by injection of CTX into the tibialis anterior (TA) muscles of 4-month-old mice. (**A**) H&E transverse sections of *Lemd* ^*+/+*^ and *Lemd2* ^*+/Gt*^ TA control muscles, and TA muscles at days 3, 4, 5, 6, and 14 after CTX injury. * necrotic myofibers found in heterozygous TA samples 3 days post-CTX. (**B**) Mean myofiber diameter at days 3, 4, 5, 6, and 14 post-CTX injury. (**C**) Cross-sectional diameters of regenerated myofibers in wild-type and *Lemd2* ^*+/Gt*^ TA muscle 6 days after CTX injury. Diameter of 100 myofibers with central nuclei were measured by ImageJ. Six samples of each genotype were analyzed. Data are presented as mean ± SD. (*p < 0.0005, **p < 0.005, ***p < 0.05, ns “not significant”).

Although *Emd* ^*–/Y*^ mice do not exhibit overt muscular dystrophy, muscle regeneration in these mice is slightly delayed [23]. Since the muscle of *Lemd2* ^*+/Gt*^ mice contains only one-half the amount of Lem2 as *Lemd2* ^*+/+*^ mice (S1B Fig.), we evaluated whether the reduced levels of Lem2 resulted in differences in muscle regeneration. Muscle injury was induced by injection of cardiotoxin (CTX) into the tibialis anterior (TA) muscle; histological analyses were carried out at five time points after CTX injection. At 3 days post-injection, there was massive inflammatory infiltration along with necrotic muscle fibers (Fig. 8A). Necrotic fibers were much more abundant in *Lemd2* ^*+/Gt*^ muscles at this time point (asterisks), suggesting that the inflammatory response might be delayed in those mice. Four days after the injection, necrotic fibers were absent, and the muscle contained small regenerating myofibers with centrally located nuclei and an extensive immune cell infiltrate. The nascent myofibers progressively increased in diameter through days 5 and 6, concomitant with a reduction in the immune cell infiltrate. Although the diameter of regenerating fibers in *Lemd2* ^*+/Gt*^ and *Lemd2* ^*+/+*^ mice was similar on days 4 and 5, the average cross-sectional diameter of regenerating myofibers at day 6 was significantly smaller in *Lemd2* ^*+/Gt*^ mice than in wild-type counterparts (Fig. 8B). The size distribution was biased towards smaller diameter myofibers in *Lemd2* ^*+/Gt*^ mice (Fig. 8C). At day 14 post-injection, these differences disappeared (Fig. 8A); the average muscle fiber diameter in *Lemd2* ^*+/Gt*^ and wild-type mice was indistinguishable. Also, the muscles in both groups of mice appeared largely regenerated, although centralized nuclei still were abundant.

In summary, with CXT-induced muscle regeneration, the *Lemd2* ^*+/Gt*^ mice had delayed removal of necrotic tissue and slower growth at the midpoint of the regenerative process, but regeneration ultimately occurred. Consistent with that finding, there was no overt muscular dystrophy phenotype in skeletal muscle of *Lemd2* ^*+/Gt*^ mice.

## Discussion

In the current study, we show that disruption of the mouse *Lemd2* gene leads to embryonic lethality by E11.5. The *Lemd2* gene-trap allele used for this analysis, instead of producing a fusion protein containing the first 296 residues of Lem2 fused to βgeo, yielded low levels of an ~32-kDa Lem2 fragment that was likely released from the fusion protein by proteolytic cleavage. Lem2 expression was evident in E8.5 embryos by western blotting. In E10.5 embryos, *Lemd2* expression was detected throughout the embryo by X-gal staining and was found in cells arising from all three germ layers. Thus, the expression of Lem2 substantially precedes that of lamins A/C, which appears only at E12 in mouse [36].

The E10.5 *Lemd2* ^*Gt/Gt*^ embryos displayed many of the hallmark morphogenetic features of organogenesis found in wild-type littermates, although several abnormalities were apparent. The mutant embryos were smaller with reduced amounts of blood, had a lower cell density in neural tissue and mesenchyme, and manifested underdevelopment of parts of the heart and neural tissue. Also, cell proliferation in the neuroepithelium cells was reduced in *Lemd2* ^*Gt/Gt*^ embryos, and apoptosis was much more frequent. It seems unlikely that anemia was the principal cause of the reduced growth of *Lemd2* ^*Gt/Gt*^ embryos: mutant mice with disruptions in genes required for definitive erythropoiesis are paler than *Lemd2* ^*Gt/Gt*^ embryos at comparable stages, yet show no size reduction [40,41]. Moreover, we rarely observed evidence of necrosis in E10.5 *Lemd2* ^*Gt/Gt*^ embryos.

The most striking phenotype of embryos at E10.5 was aberrant regulation of MAP kinase and AKT signaling pathways. In whole-embryo extracts, we observed markedly increased activation of three groups of MAP kinases—ERK1/2, JNK1/2, and p38. ERK1/2 showed the largest change, with a 7–10-fold increase. Also, we detected a 4–10-fold increase in phosphorylation of two well-studied activation sites of AKT (Thr308 and Ser473) [38]. Hyperactivation of these different signaling pathways could readily explain the embryonic lethality of *Lemd2* mutants. In particular, enhanced ERK signaling promotes apoptosis in certain cell contexts (reviewed in [42]). It is noteworthy that elevated levels of MAP kinases [21,43] and AKT/mTORC [44,45] have been reported in emerin and lamin A mouse models for cardiomyopathy and muscular dystrophy. In light of these findings and the effects of other LEM domain protein deficiencies in *C*. *elegans* [25] and *Drosophila* [26], *LEMD2* should be considered as a candidate disease gene in humans with muscle disorders.

In contrast to mice with the *Lemd2* ^*Gt/Gt*^ genotype, *Lemd2* ^*+/Gt*^ mice grew to adulthood with no overt abnormalities, even though they contained about one-half the wild-type level of Lem2 in heart and skeletal muscle. Nonetheless, there was a modest delay in CTX-induced muscle regeneration in these mice. That finding, along with the fact that Lem2 is expressed at particularly high levels in striated muscle [34], supports the possibility that Lem2 is involved in muscle homeostasis in mammals. However, a more complete evaluation of this issue will require creating a conditional knockout allele for *Lemd2* and breeding muscle-specific *Lemd2* knockout mice.

A homozygous gene-trap disruption of mouse *Lemd3* (encoding MAN1) also led to lethality by E11.5 [19,20], but the phenotype of these mutants was distinct from *Lemd2* knockout mice. At E10.5, *Lemd3* knockout embryos [19,20] were much smaller than the *Lemd2* ^*Gt/Gt*^ embryos described here. Also, the demise of *Lemd3* embryos was linked to overt defects in vasculogenesis associated with a grossly enlarged pericardium and frequent internal hemorrhages [19,20]. These aberrations were not observed in *Lemd2* ^*Gt/Gt*^ embryos. At the molecular level, the abnormalities observed in *Lemd3* knockout embryos were attributed to elevated TGF-β signaling and was accompanied by increased levels of phospho-Smad2 [19,20]. By contrast, there was no increase in the level of phospho-Smad2 in extracts of *Lemd2* ^*Gt/Gt*^ embryos. Thus, in embryos at mid-gestation, Lem2 and MAN1 regulate distinct signaling systems.

Knockdown of Lem2 in cultured C2C12 cells by siRNAs led to activation of ERK1/2, JNK1/2, p38 and AKT, similar to the results with *Lemd2* ^*Gt/Gt*^ embryos. Although silencing of emerin in C2C12 cells resulted in increased phosphorylation of the three MAP kinases, no effects on AKT were observed. These results suggest that in mice, Lem2 affects a broader range of signaling networks than emerin. Considering that Lem2 and emerin have partially overlapping functions [29], these results can explain the disparate phenotypes of the gene disruptions in mice [*i*.*e*., lack of a significant phenotype in emerin-deficient mice [23] *versus* embryonic lethality in the Lem2 mutant mice].

We have been unable to determine whether the activation of MAP kinases and AKT with loss of Lem2 in mice and C2C12 cells involves signaling pathway crosstalk (i.e., whether the increased activity of MAP kinases with loss of Lem2 is due to elevated AKT, or vice versa). We found that treatment of Lem2-silenced C2C12 cells with a MEK inhibitor to reduce activated ERK1/2 had no effect on AKT activation, whereas treatment with a PI3 kinase inhibitor to inhibit AKT caused a time-dependent oscillation in the levels of activated ERK1/2 (unpublished data). In future work it may be possible to use mutational analysis of Lem2 to shed light on this question.

The increased activation of MAP kinases and AKT resulting from the loss of Lem2 could be due to direct or indirect mechanisms. In one potential direct mechanism, Lem2 could provide a binding platform at the INM to bring signaling effectors and phosphatases into close proximity to enhance pathway inactivation. This would be analogous to the model of MAN1 in TGF-β attenuation [18]. Consistent with this possibility, most of the N-terminal nucleoplasmic domain of Lem2 (apart from the LEM domain itself) is predicted to be unstructured by the DisEMBL server, and unstructured protein domains often turn out to be docking sites for signaling regulators [46]. One potential indirect mechanism could involve a role for Lem2 in chromatin structure and gene expression. The LEM domain protein LAP2β has been reported to interact with chromatin regulators-including HDAC3-to promote gene silencing [47,48]. Lem2 could engage in similar types of mechanisms to alter programs of gene expression that influence signaling. Moreover, since the LEM domain of Lem2 interacts with BAF [30,32], the loss of a BAF binding site at the INM could alter the balance of chromatin-associated functions mediated by BAF-LEM domain interactions. Resolution of these questions should be facilitated by dissection of Lem2 domains and interacting partner proteins in future work.

## Materials and Methods

### Ethics statement

Use of mice in this project conforms to the *Guide for the Care and Use of Laboratory Animals* published by the US National Institutes of Health (NIH publication No. 85–23, revised 2011) and follows protocols approved by the Scripps Research Institute Animal Care and Use Committee (IACUC # 09–0059–02 “Organization and Functions of the Nuclear Lamina”). All surgical procedures were done after mouse anesthesia by isoflurane, as directed by the IACUC protocols.

### Generation and genotyping of *Lemd2* ^*Gt/Gt*^ mice

The ES cell line DD0639, which contains a gene-trap insertion mutation in the *Lemd2* locus (Wellcome Trust Sanger Institute, UK), was used to derive knockout mice. The resulting *Lemd2* ^*Gt*^ line was backcrossed for 10 generations onto a C57BL/6 background, and subsequently was intercrossed to obtain homozygous *Lemd2* ^*Gt/Gt*^ mice. Genotyping was performed by PCR using genomic DNA from tail biopsies or yolk sacs. We used a forward primer in intron 3 (5′-CTCCCAGGGATCCACTAACAATGG-3′) and a reverse primer located within exon 4 (5′-GCCTGCAGCCGACTCACAGC-3′); this PCR reaction yielded a 531-bp product in the wild-type allele. A second PCR reaction used forward primer 5′-CTACGGCCTGTATGTGGTGGATGAA-3′ and reverse primer 5′-GAAACCGCCAAGACTGTTACCCATC-3′ (located in βgeo sequences), yielding a 436-bp product with the mutant allele.

### Morphological and histological analysis

*Lemd2* ^*+/Gt*^ mice were intercrossed, and pregnant *Lemd2* ^*+/Gt*^ mice were sacrificed at different time points *post-coitum*. For histology, embryos were fixed in zinc-buffered formalin fixative, embedded in paraffin, cut into 10-mm sections, and stained with hematoxylin and eosin (H&E). Whole-mount X-gal staining of embryos was performed using the β-galactosidase reporter gene staining kit (Sigma) according to the manufacturer’s instructions. Sections were counterstained with Nuclear Fast Red. Slides were digitized on a Leica SCN400 slide scanner and visualized on the SlidePath Digital Image Hub. Regions of interest were captured with a screen capture tool, and images were arranged with Adobe Photoshop CS5.

### Immunofluorescence microscopy and related assays

Embryos were fixed in PBS-buffered 4% paraformaldehyde, cryoprotected by overnight incubation in 30% sucrose/PBS, and embedded in Tissue-Tek OCT (Sakura Finetek). Sagittal 8-mm sections were cut on a cryostat and mounted on Superfrost slides (Fisher Scientific). Sections were rehydrated in PBS and permeabilized for 1 h in 0.5% Triton X-100. After blocking in 3% BSA, primary antibodies were incubated overnight at 4°C followed by fluorophore-conjugated secondary antibodies. The following primary antibodies were used: mouse monoclonal anti-BrdU Alexa Fluor 488 conjugate (B35130, Invitrogen); mouse anti-class III β-Tubulin (T8578, Sigma); rabbit polyclonal anti-phospho-ERK1/2 (Thr202/Tyr204; #9101, Cell Signaling Technology (CST) were each used at a 1:100 dilution. Nuclear counterstaining was performed with Hoechst 3342. Stained tissue was analyzed on a Carl Zeiss 710 confocal scanning laser microscope. Imaging used a 63× Plan Apo objective (1.4 NA) and a pinhole setting of 1 Airy unit. Single images were used for figures, and contrast was adjusted using Adobe Photoshop CS5.

To analyze cell proliferation, pregnant females received an intraperitoneal injection of 50 μg/g body weight bromodeoxyuridine (BrdU) (Sigma-Aldrich) and sacrificed 30 min later. Embryos were isolated and processed for immunofluorescence microscopy with a primary Alexa488-conjugated monoclonal anti-BrdU antibody (B35130, Invitrogen). To analyze apoptosis, sections were prepared as described above. DNA fragmentation was detected with a transferase-mediated dUTP nick-end-labeling (TUNEL) method using a fluorescent staining kit according to the manufacturer’s instructions (Roche Diagnostics).

### RNA isolation and Northern blotting

Total RNA was extracted with Trizol reagent (Invitrogen), and poly(A)^+^ RNA was purified with the Dynabeads mRNA purification kit (Dynal). For Northern blotting, 120 μg of poly(A)^+^ RNA from whole embryos were separated on 3% formaldehyde 1% agarose gels and transferred to Hybond membranes (GE Healthcare Life Sciences). The membrane was incubated with a ^32^P-labeled RNA probe corresponding to nucleotides 707–726 of the *Lemd2* transcript in ULTRAhyb-oligo hybridization buffer (Ambion-Life Technologies).

### Cell culture and RNA interference

C2C12 cells (ATCC CRL-1772) were maintained in Dulbecco’s modified Eagle’s medium supplemented with 10% fetal bovine serum, L-glutamine, sodium pyruvate, nonessential amino acids, and antibiotics. Small interfering RNAs duplexes targeting mouse Lem2 (#1: 5′-GCUGGUCUCUGUUUCUUAA-3′; #2: 5′-GCUCAUUCACACCUGCCUU-3′), emerin (#1: 5′-ACUACUAUGAGGAGAGUUAUUUGAC-3′; #2: 5′-GCCUAAGGCAAUGCUUGUCUCCCAC-3′), and a nontargeting control siRNA (5′-ACTGTCACAAGTACCTACA-3′) were purchased from Integrated DNA Technologies. siRNAs were introduced into C2C12 cells by incubating 1.5 × 10^5^ cells (resuspended in complete medium after trypsinization) with a complex of 50 pmol siRNA and 5 μl Dharmafect 1 (Dharmacon, Lafayette, CO). After 24 h, cells were transfected a second time using the same protocol. The medium was replaced with fresh growth medium on the morning after each transfection. Cells were analyzed 72 h after first siRNA transfection.

### Immunoblot analysis

To quantify phosphorylation levels of MAPKs, AKT, and Smad2, protein lysates were prepared by dissolving whole embryos, muscle samples, or cell monolayers in SDS loading buffer containing proteinase and phosphatase inhibitor cocktails (Roche). Equal cell equivalents of total protein (20 μg) were resolved on Novex 4–20% Tris-glycine gels (Invitrogen) and transferred to nitrocellulose membranes. Membranes were blocked with 5% nonfat dry milk reconstituted in PBS with 0.1% Tween 20 (PBS/T) and incubated with the following primary antibodies diluted in PBS/T: mouse monoclonal anti-emerin (NCL-EMERIN, Novocastra; dilution 1:5000), anti-GAPDH (ab9484, Abcam; dilution 1:10000), and anti-β-galactosidase (Z3781, Promega; dilution 1:200); and rabbit polyclonal anti-Erk1/2 (#9102, CST; dilution 1:1000), anti-phospho-Erk1/2 (Thr202/Tyr204; #9101, CST; dilution 1:500), anti-p38 (#9212, CST; dilution 1:1000), anti-phospho-p38 (Thr180/Tyr182; #9211, CST; dilution 1:1000), anti-SAPK/JNK (#9252, CST; dilution 1:500), anti-phospho-SAPK/JNK (Thr183/Tyr185; #9251, CST; dilution 1:500), anti-AKT (#4691, CST, dilution 1:2000), anti-phospho-AKT (Thr308; #2965, CST; dilution 1:2000), anti-phospho-AKT (Ser473; #4060, CST, dilution 1:1000), anti-phospho-SMAD2 (Ser465/467; #138D4, CST; dilution 1:500) and anti-SMAD2 (#D43B4, CST, dilution 1:500). Antibodies against synthetic peptides of murine Lem2 (residues 149–169 and 202–221) were affinity-purified against the immunogen (Chen et al., 2006) and used at a 1:200 dilution. Following washes in PBS-T, blots were incubated with corresponding polyclonal anti-mouse and anti-rabbit secondary antibodies, horseradish peroxidase conjugated, diluted in PBS/T at a 1:5000 dilution (#115–035–003 and #111–035–003, Jackson Immunoresearch Inc.); secondary antibody binding was detected by chemiluminescence. Detection and quantitative analysis was performed with digital imaging systems (UVP; Alpha Innotech) and their software applications.

### Muscle regeneration analysis

Cardiotoxin (CTX) was injected into both tibialis anterior (TA) muscles of 12–16-week-old age- and sex-matched *Lemd2* ^*+/+*^ and *Lemd2* ^*+/Gt*^ mice. Mice were euthanized at 3, 4, 5, 6, 7 or 14 days after CTX-induced injury. The TA muscles of 6 mice per strain and per time-point were harvested and divided into two sections: one section was flash-frozen and used for protein studies, and the second section was fixed in zinc-buffered formalin fixative for H&E staining. The three muscles for each strain and time-point that showed the most consistent degeneration/regeneration patterns were selected for further analysis.

## Supporting Information

S1 Fig Lem2 expression in gene-trap embryos and adults, and normal postnatal phenotype in *Lemd2* ^*+/Gt*^ mice. (**A, B**) Levels of Lem2 determined by western blot analysis of extracts from E8.5, E10.5, and E13.5 embryos of the indicated genotypes (**A**) and of extracts from three separate *Lemd2* ^*+/+*^ and *Lemd2* ^*+/Gt*^ adult TA muscles (**B**). A Lem2/βgeo fusion protein (~175 kD) was not detected in either embryonic or adult muscle samples. The 32-kDa Lem2 fragment was detected only in *Lemd2* ^*+/Gt*^ and *Lemd2* ^*Gt/Gt*^ embryonic samples (left panel). Anti-βgal antibodies recognized a 115-kD band, the predicted molecular weight for β-galactosidase, in both embryo and adult samples. GAPDH was the loading control. (**C**) Graph showing the relative intensities of the Lem2 and 32-kDa Lem2 fragment bands in extracts from E10.5 *Lemd2* ^*+/Gt*^ and *Lemd2* ^*Gt/Gt*^ embryos, as compared to the intensity of wild-type Lem2 (100%) in extracts of *Lemd2* ^*+/+*^ embryos (*n* = 3 for +/+, 10 for +/Gt, 5 for Gt/Gt). (**D**) Body weight of *Lemd2* ^*+/Gt*^ mice (grey bars) compared to that of age-/sex-matched wild-type mice (white bars). Bars represent mean ± SD (*n* = 10 in each group). No deaths or abnormal behavioral phenotypes were seen in a *Lemd2* ^*+/Gt*^ mouse population (*n* > 100) for up to 1 year (not shown). (**E**) Histological analysis of skeletal and cardiac muscle from wild-type (control) or heterozygous (*Lemd2* ^*+/Gt*^) mice with H&E and Gomori’s trichrome staining. Localization of myonuclei was normal, and fibrosis-positive areas (blue) were not observed. Bars: 25 μm.
(TIF)

S2 Fig Abnormal phenotypes including craniofacial development defects observed in a minor fraction of *Lemd2* ^*Gt/G*t^ embryos. Lateral (left and right) views of whole embryos at E10.5. Compared to development in the wild-type embryo (left), a minor fraction (~10%) of the *Lemd2* ^*Gt/Gt*^ embryos (right) exhibited a lack of the telencephalic vesicles and an abnormal hindbrain (arrows). Several intracranial hemorrhages also were observed (arrowheads), as well as an open neural tube at the posterior region of the embryo (asterisk). Posterior and frontal views show magnified craniofacial structures. Posterior view: the mutant embryo exhibited collapsed hindbrain and midbrain regions, lacking the lumen of the neural tube (arrows). Frontal view: the *Lemd2* ^*Gt/Gt*^ embryo exhibited an open neural tube at the midbrain (arrow) and forebrain (arrowhead) and lack of telencephalic vesicles. The frontonasal process formation appeared normal. f, forebrain; fnp, frontonasal process; h, hindbrain; m, midbrain; tv, telencephalic vesicle.
(TIF)

S3 Fig Histological analysis of multiple organs from *Lemd2* ^*Gt/G*t^ embryos. Sagittal sections of *Lemd2* ^*+/+*^ (left) and *Lemd2* ^*Gt/Gt*^ (right) E10.5 embryos stained with H&E. Mutant embryos exhibited lower mesenchymal cell density (**A**) and somites with a partially collapsed appearance (**B**). (**C**) The Wolffian duct (WD) and the metanephric mesenchyme (MM) appeared normal in the *Lemd2* ^*Gt/Gt*^ embryos. The distal forelimb (**D**) and hindlimb (**F**) buds showed the formation of a normal multi-layered AER (apical ectodermal ridge) in the mutants (arrows). (**E**) Retina (R) and lens (L) development appeared grossly normal in the *Lemd2* ^*Gt/Gt*^ embryos although the density of neuroepithelial cells in the retina appeared to be lower. (**G**) Pharyngeal arches in the mutant embryos were grossly normal but smaller in size. (**H**) Dorsal root ganglia (brackets) appeared misorganized and had a lower cell density in the *Lemd2* ^*Gt/Gt*^ embryos. Bars: 20 μm (A-D, F, H); 50 μm (E, G).
(TIF)
