# Supplementary figures and images for: Nuclear Envelope Protein Lem2 is Required for Mouse Development and Regulates MAP and AKT Kinases
Source: PLoS One. 2015 Mar 19;10(3):e0116196. doi: 10.1371/journal.pone.0116196 (PMC4366207; doi:10.1371/journal.pone.0116196)

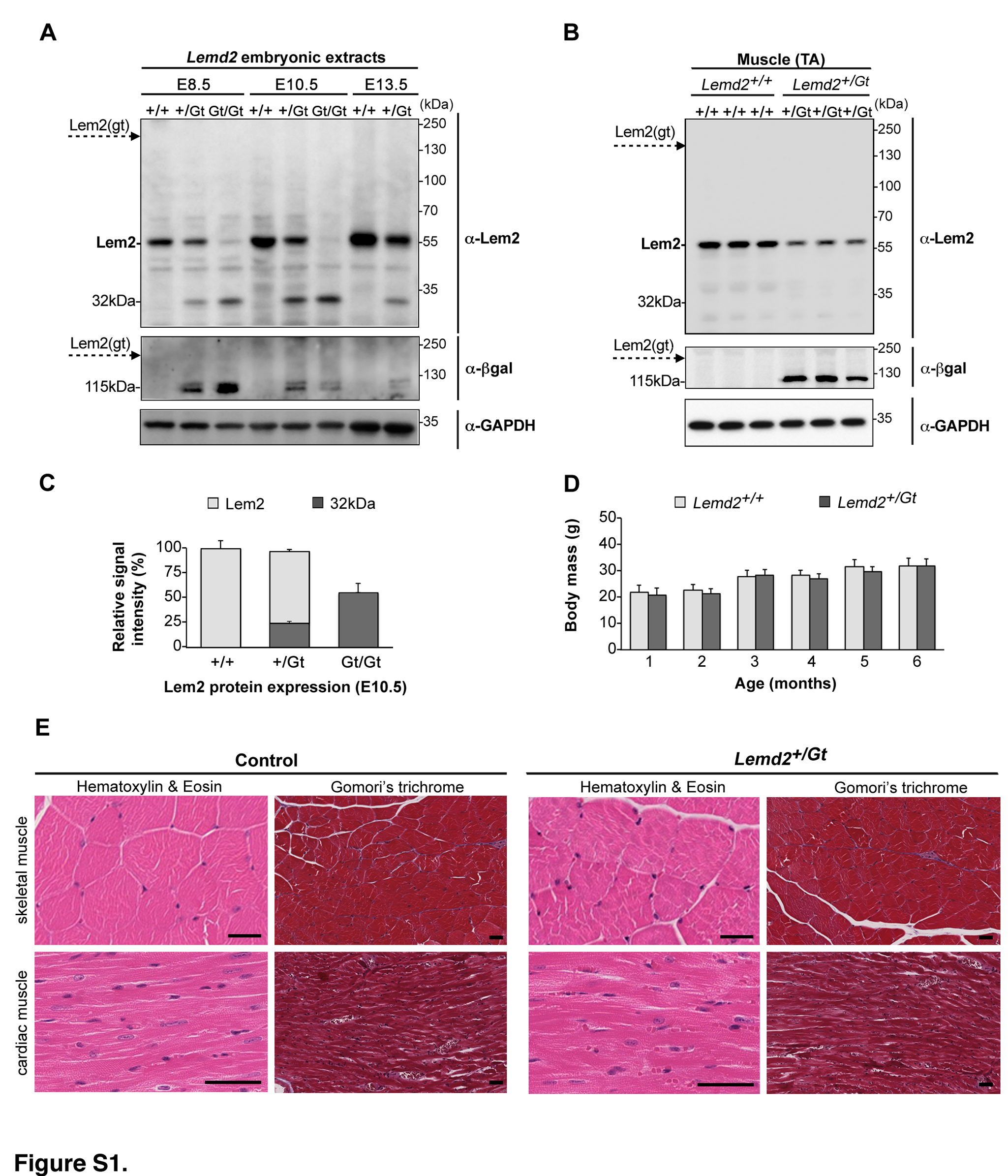

Supplement: S1 Fig — (A, B) Levels of Lem2 determined by western blot analysis of extracts from E8.5, E10.5, and E13.5 embryos of the indicated genotypes (A) and of extracts from three separate Lemd2 +/+ and Lemd2 +/Gt adult TA muscles (B). A Lem2/βgeo fusion protein (~175 kD) was not detected in either embryonic or adult muscle samples. The 32-kDa Lem2 fragment was detected only in Lemd2 +/Gt and Lemd2 Gt/Gt embryonic samples (left panel). Anti-βgal antibodies recognized a 115-kD band, the predicted molecular weight for β-galactosidase, in both embryo and adult samples. GAPDH was the loading control. (C) Graph showing the relative intensities of the Lem2 and 32-kDa Lem2 fragment bands in extracts from E10.5 Lemd2 +/Gt and Lemd2 Gt/Gt embryos, as compared to the intensity of wild-type Lem2 (100%) in extracts of Lemd2 +/+ embryos (n = 3 for +/+, 10 for +/Gt, 5 for Gt/Gt). (D) Body weight of Lemd2 +/Gt mice (grey bars) compared to that of age-/sex-matched wild-type mice (white bars). Bars represent mean ± SD (n = 10 in each group). No deaths or abnormal behavioral phenotypes were seen in a Lemd2 +/Gt mouse population (n > 100) for up to 1 year (not shown). (E) Histological analysis of skeletal and cardiac muscle from wild-type (control) or heterozygous (Lemd2 +/Gt) mice with H&E and Gomori’s trichrome staining. Localization of myonuclei was normal, and fibrosis-positive areas (blue) were not observed. Bars: 25 μm. (TIF) [file pone.0116196.s001.tif]

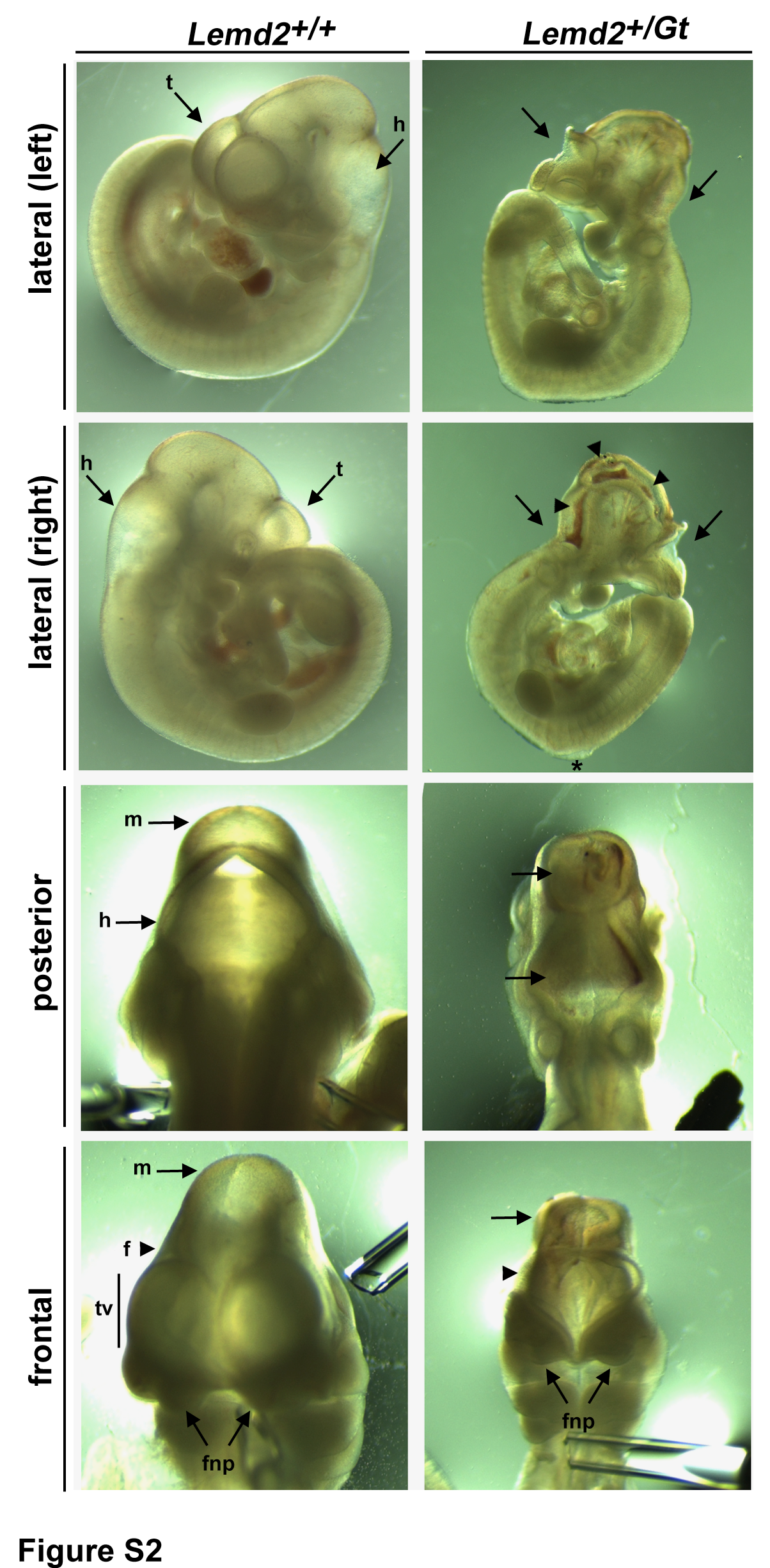

Supplement: S2 Fig — Lateral (left and right) views of whole embryos at E10.5. Compared to development in the wild-type embryo (left), a minor fraction (~10%) of the Lemd2 Gt/Gt embryos (right) exhibited a lack of the telencephalic vesicles and an abnormal hindbrain (arrows). Several intracranial hemorrhages also were observed (arrowheads), as well as an open neural tube at the posterior region of the embryo (asterisk). Posterior and frontal views show magnified craniofacial structures. Posterior view: the mutant embryo exhibited collapsed hindbrain and midbrain regions, lacking the lumen of the neural tube (arrows). Frontal view: the Lemd2 Gt/Gt embryo exhibited an open neural tube at the midbrain (arrow) and forebrain (arrowhead) and lack of telencephalic vesicles. The frontonasal process formation appeared normal. f, forebrain; fnp, frontonasal process; h, hindbrain; m, midbrain; tv, telencephalic vesicle. (TIF) [file pone.0116196.s002.tif]

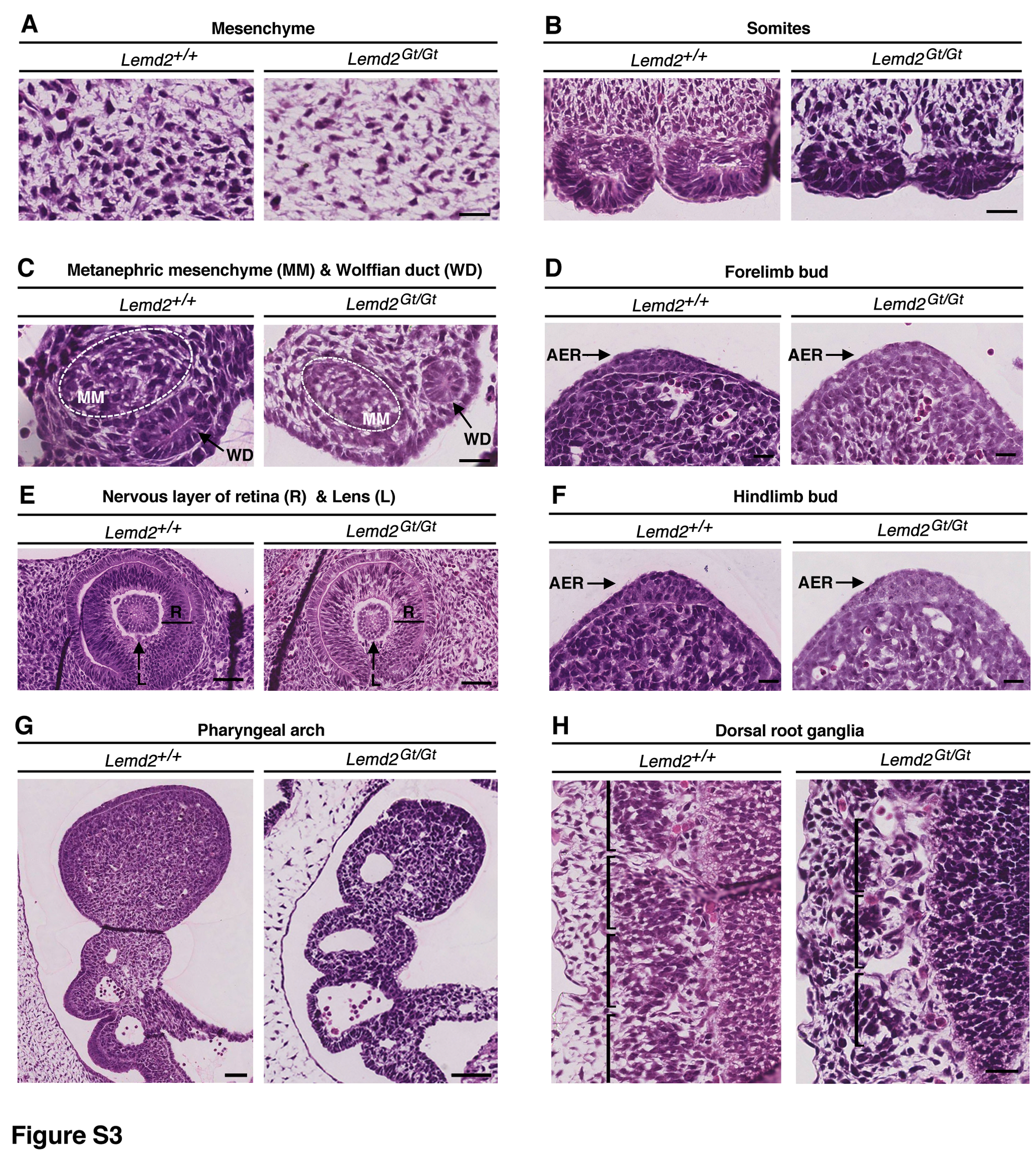

Supplement: S3 Fig — Sagittal sections of Lemd2 +/+ (left) and Lemd2 Gt/Gt (right) E10.5 embryos stained with H&E. Mutant embryos exhibited lower mesenchymal cell density (A) and somites with a partially collapsed appearance (B). (C) The Wolffian duct (WD) and the metanephric mesenchyme (MM) appeared normal in the Lemd2 Gt/Gt embryos. The distal forelimb (D) and hindlimb (F) buds showed the formation of a normal multi-layered AER (apical ectodermal ridge) in the mutants (arrows). (E) Retina (R) and lens (L) development appeared grossly normal in the Lemd2 Gt/Gt embryos although the density of neuroepithelial cells in the retina appeared to be lower. (G) Pharyngeal arches in the mutant embryos were grossly normal but smaller in size. (H) Dorsal root ganglia (brackets) appeared misorganized and had a lower cell density in the Lemd2 Gt/Gt embryos. Bars: 20 μm (A-D, F, H); 50 μm (E, G). (TIF) [file pone.0116196.s003.tif]
